# Successful Diagnosis of a SMARCA4-Deficient Undifferentiated Tumor via Endobronchial Ultrasound-Guided Fine Needle Aspiration and Endobronchial Biopsies

**DOI:** 10.7759/cureus.76649

**Published:** 2024-12-30

**Authors:** Luis F Meza, Mark Bowling, Soban Farooq, Bryan K Dunn

**Affiliations:** 1 Pulmonary and Critical Care Medicine, Brody School of Medicine, East Carolina University, Greenville, USA; 2 Pulmonary and Critical Care, Brody School of Medicine, East Carolina University, Greenville, USA; 3 Internal Medicine and Pathology, Brody School of Medicine, East Carolina University, Greenville, USA

**Keywords:** bronchoscopy, ebus-tbna, non small cell lung cancer, smarca, smarca deficient

## Abstract

Lung cancer is the third most prevalent cancer, following breast cancer in women and prostate cancer in men. However, it remains the leading cause of cancer-related mortality. As treatment options have advanced, the significance of accurate diagnosis has increased, enabling targeted and more personalized therapeutic treatments. SMARCA4-deficient thoracic tumors are a relatively new classification of mediastinal lung cancers that are known to be difficult to diagnose with conventional endobronchial ultrasound-transbronchial needle aspiration (EBUS-TBNA). Newer modalities such as endobronchial ultrasound with cryobiopsy have emerged, and studies have indicated that they offer increased diagnostic yield when coupled with conventional EBUS-TBNA. Here, we present a case in which EBUS-TBNA alone produced sufficient tissue volume for immunohistochemistry at NeoGenomics (Fort Myers, FL), leading to a successful diagnosis.

## Introduction

The American Cancer Society estimated that in the year 2024, there would be 234,580 new cases of lung cancer, leading to 125,070 deaths in both men and women [1,2]. As a result, prompt diagnosis to guide timely and efficient treatment remains of utmost importance. Societal guidelines still recommend endobronchial ultrasound-transbronchial needle aspiration (EBUS-TBNA) as the gold standard for lung cancer staging [3,4,5].

Thoracic SMARCA4-deficient tumors were first included in the World Health Organization’s (WHO's) 2015 classification of thoracic neoplasms as a distinct category to facilitate tailored therapeutic approaches [6]. In 2021, the name was changed to SMARCA4-deficient undifferentiated tumors (SMARCA4-UTs) according to the WHO classification of thoracic tumors, to incorporate the findings of more recent studies [7].

Our literature search revealed that although SMARCA4-undifferentiated tumors have been diagnosed using a combination of cryogenic biopsy with bronchoscopy and EBUS-TBNA or surgical biopsy, no successful diagnosis using only EBUS-TBNA has been made [8,9]. Here, we report a rare case of a SMARCA4-UT successfully diagnosed by EBUS-TBNA using a 22-gauge needle.

## Case presentation

Our patient was a 67-year-old male with a past medical history of tobacco dependence (50 pack years), post-prostatectomy prostate cancer status, hypertension, and hyperlipidemia. He presented to our outpatient clinic at the request of his oncologist owing to an abnormal computed tomography (CT) of the chest.

**Figure 1:**
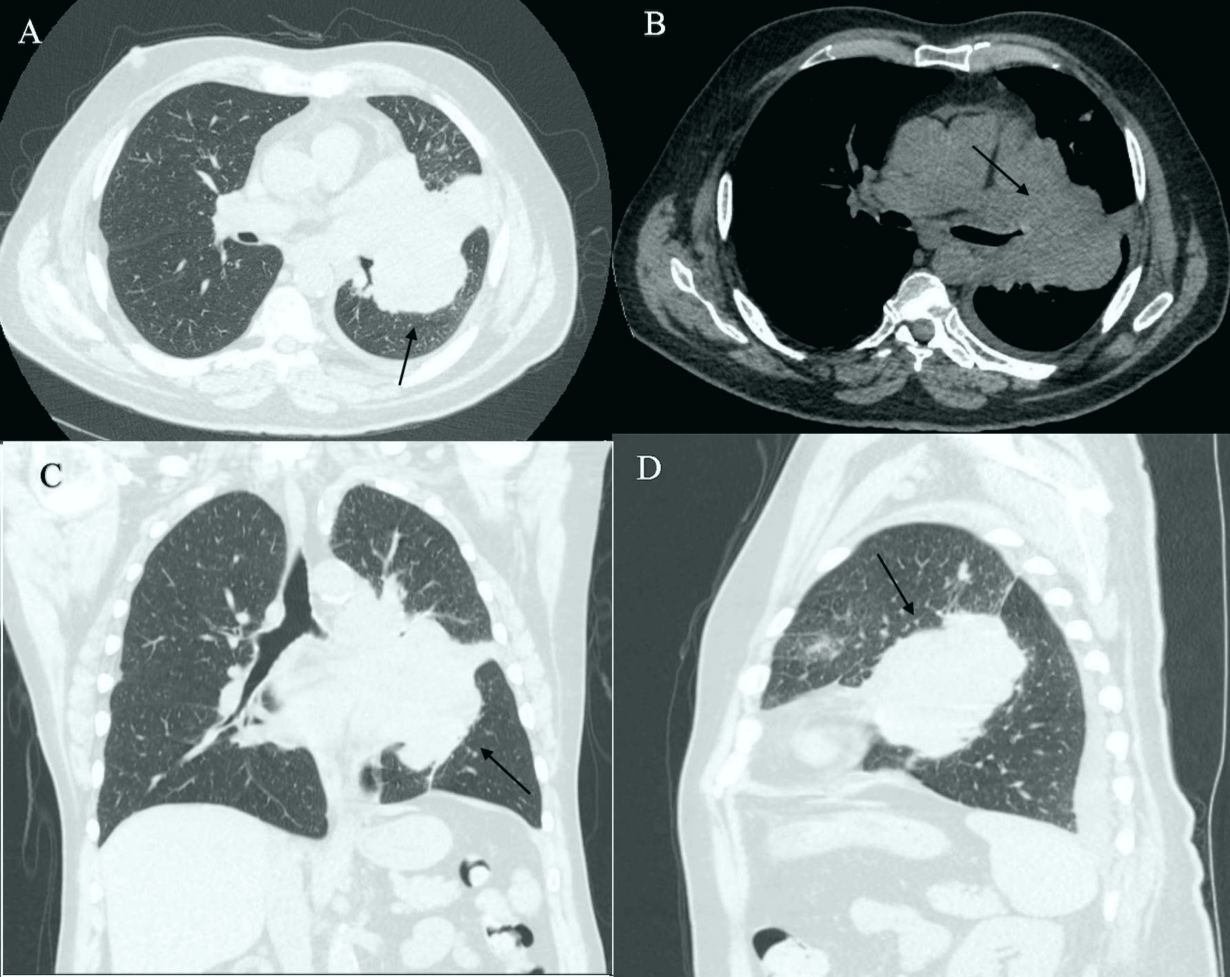
CT chest without contrast before the procedure. (A) Lung window view showing left hilar mass (arrow), (B) mediastinal view demonstrating left hilar adenopathy (arrow), (C) coronal view of left hilar mass (arrow), and (D) sagittal view of left hilar mass (arrow).

Our patient reported worsening shortness of breath, particularly with exertion, accompanied by a worsening cough with sputum production and subjective weight loss. A chest CT without intravenous contrast was performed, revealing a new mass encasing the left hilum, measuring up to 10 cm and extending into the left lower lobe bronchi and left lower lobe, with extensive hilar and mediastinal lymphadenopathy also noted (Figures 1A-1D).

**Figure 2:**
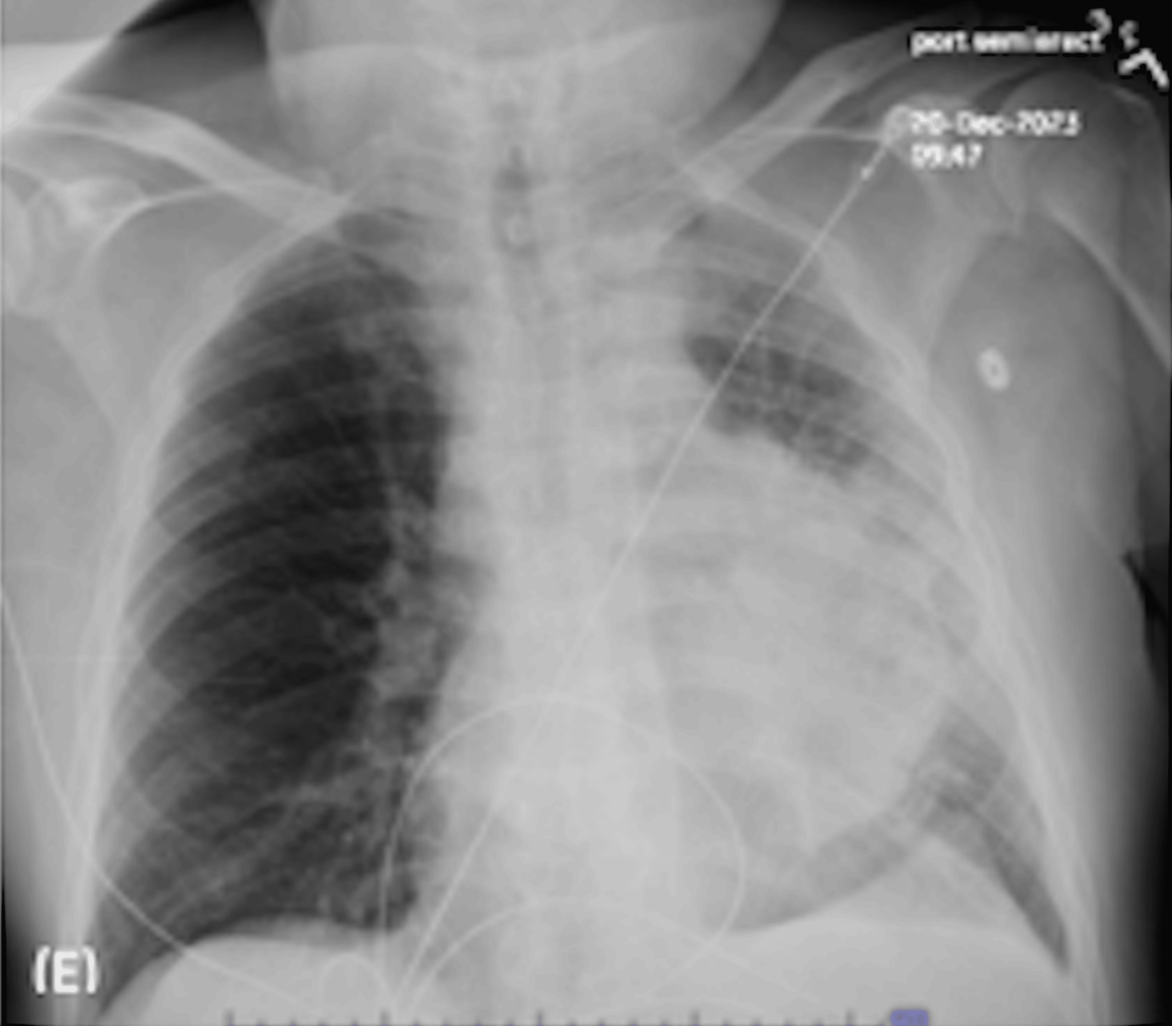
Post-procedure chest X-ray.

The decision was made to perform a bronchoscopy with biopsies. Preoperative labs, including a complete blood count, metabolic panel, and coagulation studies, revealed no abnormalities. The patient underwent bronchoscopy under general anesthesia, and initial airway inspection starting from the right lobe revealed inflamed mucosa with thick mucoid secretions at the right main stem, right upper lobe, right bronchus intermedius, and down the right middle lobe, and lower lobe. Left lung airway inspection also revealed inflamed mucosa from the left main stem down to the left lower lobe. The left upper lobe was 90% occluded with a large endobronchial lesion surrounded by inflamed mucosa, and the lingula showed inflamed mucosa with extrinsic compression proximally. Endobronchial biopsies (EBBx) at the lesion using forceps were performed, and tissue was placed for touch prep slides for rapid on-site evaluation (ROSE). Additional tissue was placed in saline for final pathology review. Using a curvilinear probe endobronchial ultrasound bronchoscope (EBUS) and a 22-gauge needle, transbronchial needle aspiration (TBNA) was performed at the lesion and evaluated via ROSE for acceptable sample quality. EBUS-TBNA was then performed, starting at the N3 lymph node, with biopsies taken from stations 4R (passes 4-5), 7 (passes 6-8), and 10L (passes 1-3), with sample quality confirmed via ROSE. Post-procedure chest X-ray was assessed and did not show a pneumothorax (Figure 2). 

**Figure 3:**
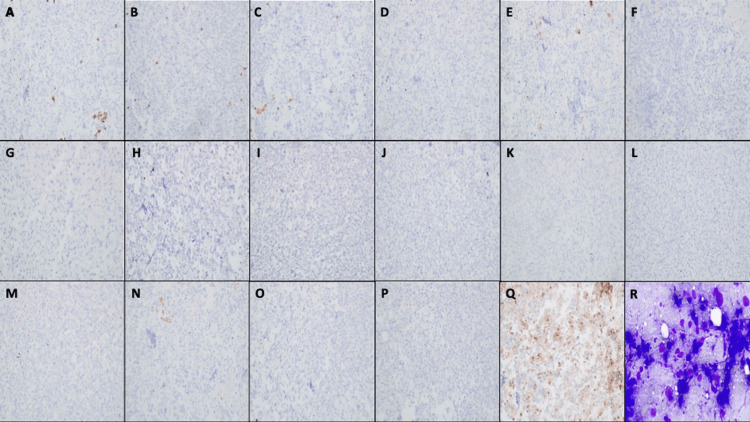
Initial negative immunochemistry staining and histochemical cytology stain (Diff Quick Stain [R]) prompting secondary tests at 300x-400x magnification (A) Negative CK8/18 (cytokeratin 8/18: cellular wall proteins); (B) negative CK5/6 (cytokeratin 5/6: stains for epithelial cancer cells); (C) negative CK7 (cytokeratin 7: type II keratin that stains adenocarcinomas of the salivary glands, lung, breast, ovary, endometrium, bladder, and thymus); (D) negative CK20 (cytokeratin 20: expressed in the gastrointestinal [GI] epithelium, urothelium, and Merkel cells but coupled with CK7, a positive predictor of lung cancer); (E) negative B72.3 (a monoclonal antibody to detect the presence of the tumor-associated glycoprotein in identifying adenocarcinomas); (F) negative S100 (a family of proteins that bind calcium, used to identify cancerous tissue); (G) negative Mart-1 (recognizes a pre-melanosomal antigen in cells of melanocyte lineage, used to detect melanoma); (H) negative HMB45 (antibody against the protein Pmel17/gp100, specific for melanoma); (I) negative SMA (smooth muscle actin, found in cells of myoepithelial origin); (J) negative Desmin (a protein found in cardiac, skeletal, and smooth muscle, used to detect various tumors such as myosarcomas); (K) negative WT-1 (antibody detection for Wilms tumor protein, a transcription factor involved in cell growth, differentiation, and apoptosis, important for cancer prognosis); (L) negative Calretinin (an antibody detecting the presence of the calcium-binding protein, expressed in many cancer cells); (M) negative Inhibin (a dimeric glycoprotein consisting of alpha and beta subunits, mainly produced in the gonads, used to detect stromal and sex chord tumors); (N) negative CD31 (stain identifying endothelial cells, confirming tumors of vascular origin); (O) negative CD117 (a tyrosine kinase receptor antibodies, identifying a wide range of cancers); (P) negative DOG1 (monoclonal antibody mainly used for identifying GI stromal tumors); (Q) negative CD45 (protein on human leukocytes, used to identify immune-related tumors); and (R) FNA Diff-Quick Staining rapid review.

**Figure 4:**
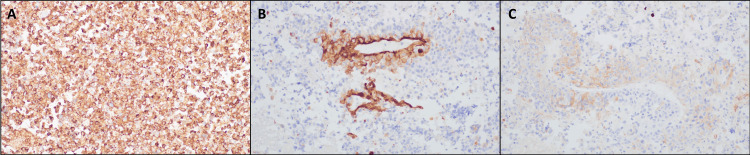
Secondary immunohistochemical staining revealing SMARCA4 deficiency at 400x magnification. (A) Negative CD34 (a 110 kDa glycosylated transmembrane protein, primarily used as a biomarker for hematopoietic cells, which can identify myeloid, lymphoid, and spindle cell cancers); (B) negative Caldesmon (a protein that binds calcium, calmodulin, tropomyosin, and actin to regulate smooth muscle contraction. Useful for distinguishing smooth muscle cell lesions [positive] from myofibroblastic cell lesions [slightly to no positivity]); (C) negative BerEp4 (an antibody to cell membrane glycoproteins, sensitive and specific for lung adenocarcinoma [positive] vs. mesothelioma [negative]).

Initial pathologist review of the EBBx and EBUS-TBNA lesion samples, before immunohistochemistry staining, revealed malignant cells with eccentric nuclei, granular chromatin, prominent nucleoli, and moderate cytoplasm. The findings appeared similar to those obtained via concurrent EBUS-TBNA on the biopsies performed at the lymph nodes. Initial immunohistochemistry staining performed on the samples tested negative for CK8/18, CK5/6, CK7, CK20, B72.3, S100, Mart-1, HMB45, SMA, Desmin, WT-1, Calretinin, Inhibin, CD31, CD117, DOG1, and CD45. Histochemical cytology with fine needle aspiration (FNA) using Diff-Quick stain was also negative (Figures 3A-3R). In addition, the tissue samples tested positive for CD34, focal Caldesmon, and focal BerEp4 (Figures 4A-4C).

SMARCA4 immunohistochemistry was performed at NeoGenomics (Fort Myers, FL), with appropriate controls. The lack of staining for SMARCA4 on the cell block obtained from the EBUS-TBNA of the lymph nodes and the lesion, combined with the results of the other immunohistochemical analyses, was sufficient to confirm a diagnosis of thoracic SMARCA4-deficient undifferentiated tumor. To confirm the diagnosis, SMARCA4 immunohistochemistry was also performed on the EBBx combined cell block.

## Discussion

In our case, EBUS-TBNA alone, using a 22-gauge needle, provided sufficient material for immunohistochemical and molecular testing to diagnose a SMARCA4-deficient undifferentiated tumor in a patient with a large left mediastinal mass. There have been previous case reports in which EBUS-TBNA failed to diagnose this specific malignancy. Takemura et al. reported a case where EBUS-cryobiopsy was required after a failed attempt to stage a SMARCA4-UT with EBUS-TBNA [9]. Zhang et al. also described a case where EBUS-FNA required additional EBUS-cryobiopsy to assist with diagnosis [10]. Additionally, Kwon and Jang reported a case in which EBUS-TBNA failed twice, and a mediastinoscopy with full lymph node removal was required for the final diagnosis [11].

SMARCA4-deficient tumors are considered notoriously difficult to diagnose using FNA of endobronchial lesions along with EBUS-TBNA, in part because of the relatively low tissue volume obtained by this modality [4]. As of 2021, fewer than 100 cases of this specific cancer had been reported, and diagnosed using various modalities [12]. Is this enough to suspect SMARCA4 tumors in every case of fast-growing malignancy, potentially necessitating more invasive procedures initially to obtain a sufficient tissue sample?

Given this, our discussion raises the question: Why did these other authors require more invasive procedures when our team was able to accomplish this with a single EBUS-TBNA? One possible reason was the equipment used - specifically, the gauge of the needle. In a study by Takemura et al., the authors used a 25-gauge needle, versus the 22-gauge needle used at our institution, for EBUS-TBNA procedures. Although their initial TBNA passes did reveal malignant cells, they required cryobiopsies for final diagnosis [9]. Was this small difference in needle size sufficient to obtain an adequate sample volume for SMARCA4 immunohistochemical staining?

Unfortunately, needle gauge was rarely reported in the cases that initially used EBUS-TBNA and were reviewed for this case report. Most cases described the patients undergoing surgical resections or lymphadenectomies as the primary sources of tissue [13,14,15].

There have been head-to-head comparisons of diagnostic yields between different gauge needles. Sakai et al. showed no difference in diagnostic yield for malignancy between a 22-gauge and 25-gauge needle (74.5% and 75.5%, respectively, *P* = 0.37). However, they did observe a significant increase in the quantity of total malignant cells per volume (median 626 [range: 5-4200] vs. 400 [range: 5-1652]) [16]. Consistent with this, a small study at St. Marianna University School of Medicine compared 22-gauge and 25-gauge needles, concluding that there was no difference in diagnostic yield for lung cancer. However, tissue volumes were inadequate for diagnosing sarcoidosis, indicating the superiority of the 22-gauge needle in terms of tissue quantity [17].

Second, we sought to determine whether there was a difference in technique while performing the biopsy itself that could have changed the results. Although bronchoscopy lab protocols vary by institution, the standard EBUS-TBNA technique used has checkpoints and quality metrics that must be achieved for it to be an acceptable and reliable procedure [18].

Currently, EBUS-TBNA is the gold standard for the staging of non-small cell lung cancer [19]. The evolving classifications of lung cancer call for different techniques for diagnosis. Endobronchial ultrasound cryobiopsy has emerged as a viable add-on procedure that can assist EBUS-TBNA. In an open-label randomized controlled trial, Fan et al. compared EBUS-TBNA coupled with transbronchial cryobiopsy versus EBUS-TBNA alone. Combining both modalities yielded a significant increase in diagnostic yield in mediastinal malignancies [8]. The combination was also found to have a favorable safety profile, hinting toward a combined approach being a valid first-line diagnostic tool. Unfortunately, new techniques are usually delayed from being readily available owing to reasons related to cost and training.

Our case showed that sufficient viable tissue samples can be obtained using EBUS-TBNA to diagnose a SMARCA4-deficient malignancy, keeping in consideration that a needle gauge is important for the much-needed volume. Until newer techniques such as EBUS-cryobiopsy are standardized, the emphasis should still be on what is available for the clinician and safe for the patient. EBUS-TBNA remains a reliable method, especially when performed with a larger bore needle. Our case adds to the limited other reports of successful SMARCA4-deficient tumor diagnosis using EBUS-TBNA alone.

## Conclusions

Our case showed that sufficient viable tissue samples can be obtained using EBUS-TBNA to diagnose a SMARCA4-deficient malignancy, keeping in consideration that a needle gauge is important for the much-needed volume. Until newer techniques such as EBUS-cryobiopsy are standardized, the emphasis should still be on what is available for the clinician and safe for the patient. EBUS-TBNA remains a reliable method, especially when performed with a larger bore needle. Our case adds to the limited other reports of successful SMARCA4-deficient tumor diagnosis using EBUS-TBNA alone.
